# Non-invasive detection of DNA methylation states in carcinoma and pluripotent stem cells using Raman microspectroscopy and imaging

**DOI:** 10.1038/s41598-019-43520-z

**Published:** 2019-05-07

**Authors:** Ruben Daum, Eva M. Brauchle, Daniel Alejandro Carvajal Berrio, Tomasz P. Jurkowski, Katja Schenke-Layland

**Affiliations:** 1 Department of Women’s Health, Research Institute for Women’s Health, Eberhard-Karls-University Tübingen, Silcherstr. 7/1, 72076 Tübingen, Germany; 2 The Natural and Medical Sciences Institute (NMI) at the University of Tübingen, Markwiesenstr. 55, 72770 Reutlingen, Germany; 3 Department of Biochemistry, Institute of Biochemistry and Technical Biochemistry, University of Stuttgart, Allmandring 31, 70569 Stuttgart, Germany; 4 Department of Medicine/Cardiology, Cardiovascular Research Laboratories, David Geffen School of Medicine at UCLA, 675 Charles E. Young Drive South, MRL 3645 Los Angeles, CA USA

**Keywords:** Super-resolution microscopy, Epigenetic memory, Embryonic stem cells, Raman spectroscopy

## Abstract

DNA methylation plays a critical role in the regulation of gene expression. Global DNA methylation changes occur in carcinogenesis as well as early embryonic development. However, the current methods for studying global DNA methylation levels are invasive and require sample preparation. The present study was designed to investigate the potential of Raman microspectroscopy and Raman imaging as non-invasive, marker-independent and non-destructive tools for the detection of DNA methylation in living cells. To investigate global DNA methylation changes, human colon carcinoma HCT116 cells, which were hypomorphic for DNA methyltransferase 1, therefore showing a lower global DNA methylation (DNMT1^−/−^ cells), were compared to HCT116 wildtype cells. As a model system for early embryogenesis, murine embryonic stem cells were adapted to serum-free 2i medium, leading to a significant decrease in DNA methylation. Subsequently, 2i medium -adapted cells were compared to cells cultured in serum-containing medium. Raman microspectroscopy and imaging revealed significant differences between high- and low-methylated cell types. Higher methylated cells demonstrated higher relative intensities of Raman peaks, which can be assigned to the nucleobases and 5-methylcytosine. Principal component analysis detected distinguishable populations of high- and low-methylated samples. Based on the provided data we conclude that Raman microspectroscopy and imaging are suitable tools for the real-time, marker-independent and artefact-free investigation of the DNA methylation states in living cells.

## Introduction

DNA methylation and de-methylation are involved in gene expression, disease mechanisms and reactions to environmental substances. It is assumed that the cellular memory is mainly inherited by DNA methylation^1^. In the DNA methylation process, methyl groups are added to cytosine within CpG dinucleotides. The reaction is catalyzed by DNA methyltransferases (DNMT)^2,3^. There are three catalytically active DNMTs in mammalians. DNMT1 maintains methylation patterns by methylating the newly synthesized DNA strand. DNMT3a and DNMT3b are required for *de novo* DNA methylation^4–6^. As methylation of promotor regions typically represses gene transcription, most of the DNA methylation-related consequences are genomic imprinting and inactivation of the X chromosome in female mammals^7^. Early embryogenesis is marked by dramatic changes in DNA methylation. After fertilization, DNA methylation in the genome becomes erased over several DNA replication cycles involving both active and passive demethylation^8^. Around implantation, DNMT3a and DNMT3b are expressed at high levels to form the normal embryonic methylation patterns^9^. In the blastocyst stage, high global levels of DNA methylation are detected^10^. Tracking of these massive changes in global DNA methylation could provide new insights about early embryogenesis. After the blastocyst status, the global DNA methylation levels do not dramatically change any more during differentiation^11–13^. However, it was shown that during carcinogenesis, in most cancers, a site-specific DNA hypermethylation and a global DNA hypomethylation takes place^14–16^. In the case when promotors of tumor suppressor genes get hypermethylated, the genes are switched off^17,18^. Global hypomethylation in turn leads to genome instability and activation of transposable elements and oncogenes^17^. It has been estimated that 70% of all cancers lead to a reduced global DNA methylation, 18% with no change, and 12% with an increased DNA methylation relative to the adjacent normal tissue^19^. This overall change of DNA methylation could serve as a valid biomarker for cancer.

There are various approaches to detect and analyze global DNA methylation. A well-established method is immunofluorescence (IF) staining based on the use of an anti-5-methylcytosine (5mC) antibody and a secondary antibody labeled with a fluorescent dye. The method offers a straightforward visualization of methylated DNA^20^. Furthermore, to study global DNA methylation, an enzyme-linked immunosorbent assay (ELISA) based on anti-5mC can be performed. Liquid chromatography-mass spectrometry is also commonly used, yet it requires sample preparation and expensive machines to measure the DNA methylation level^21^. One of the currently most widely used techniques to assess DNA methylation is the bisulfite conversion. The DNA is treated with sodium bisulfite, which deaminates non-methylated cytosines, converting them into uracils, whereas the treatment does not change methylated cytosine^22^. By comparing the sequences of converted and unconverted DNA, it is possible to identify methylated sites. However, all these methods are invasive and potentially create artifacts as they require fixation and staining procedures, cell lysis or DNA isolation. So far, there is no appropriate method established that allows the online monitoring of global DNA methylation changes in living cells.

A promising tool for online monitoring of living cells and tissues is Raman microspectroscopy as it is a non-invasive and marker-independent technique based on light scattering of the illuminated material^23^. In the last decade, Raman spectroscopy has become a method of interest for the field of biomedical research^24,25^. It is a time-saving alternative to other methods investigating biological systems such as fluorescence imaging^26^. Moreover, it allows the analysis of biological processes within living cells. The Raman measurement obtains signals from proteins, lipids, nucleic acids, carbohydrates and inorganic crystals, which enables to identify and distinguish cell phenotypes and tissues based on their individual biochemical signature^23^. The detection of DNA methylation using Raman microspectroscopy is not yet established. Some studies investigating DNA methylation were performed using surface-enhanced Raman spectroscopy (SERS)^27–29^. However, no investigations on living cells have been performed to date.

In the present study, we used Raman microspectroscopy and principle component analysis (PCA) to identify Raman shifts that can indicate global DNA methylation changes in living cells. Two cell types that differ in their global DNA methylation level were measured and compared with each other. Genetic modified human colon cancer cells that are DNMT1 hypomorph (DNMT1^−/−^ cells) were compared to HCT116 wildtype (WT) cells^30^. The DNMT1^−/−^ cells still retain some DNMT1 activity, yet much reduced, leading to a decreased genome wide methylation level. As a model system to investigate global DNA methylation changes in early embryogenesis, murine embryonic stem cells (mESC) were utilized. mESCs were cultured in serum-containing medium or they were adapted to serum-free 2i medium, leading to a significant decrease in DNA methylation^31^. The aim of the study was to investigate if Raman microspectroscopy and imaging are capable to detect the different global DNA methylation levels in both cell types. IF staining and 5mC ELISA were used to quantify the global DNA methylation in the cells. These routine methods served as reference in order to verify the results from the Raman measurements.

## Results

### 5mC and methylated DNA show significantly increased Raman bands

Before measuring DNA methylation within cells, cytidine and 5mC as well as methylated and non-methylated DNA standards were investigated with Raman microspectroscopy (Suppl. Fig. 1). The Raman spectra of 5mC and methylated DNA revealed increased signals in the region of 1331–1335 cm^−1^ (Suppl. Fig. 1a, b). Furthermore, methylated DNA showed significantly increased Raman bands at 1335 cm^−1^ (p < 0.05), 1379 cm^−1^ (p < 0.05) and 1579 cm^−1^ (p < 0.01) (Suppl. Fig. 1c).

### DNMT1-deficient cells reveal a decreased global DNA methylation

**Figure 1:**
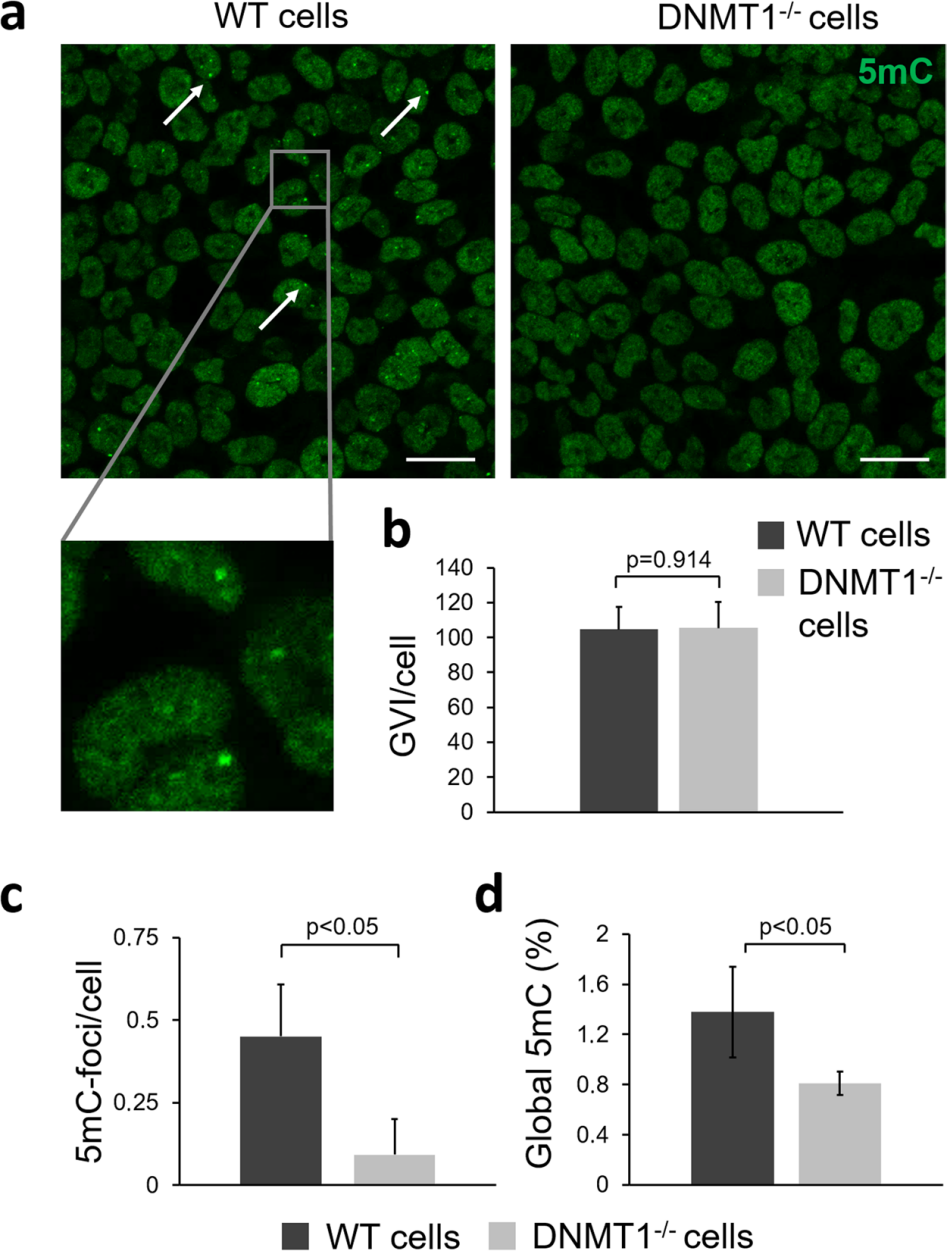
Investigation of global DNA methylation in HCT116 WT and DNMT1^−/−^ cells. (**a**) Immunofluorescence staining of 5-methylcytosine (5mC) in WT and DNMT1^−/−^ cells. WT cells show various dense 5mC-foci within the nucleus indicated by white arrows. Scale bars equal 20 µm. (**b**,**c**) Semi-quantitative fluorescence intensity analysis of 5mC-stained cells. WT cells show significant more 5mC-foci/cell than DNMT1^−/−^ cells. Two-tailed *t*-test, n = 3. (**d**) Global 5mC level in WT cells are significantly higher than in DNMT1^−/−^ cells. Two-tailed *t*-test, n = 3.

Global DNA methylation of the human colon cancer cell line HCT116 was studied. Wildtype (WT) cells and DNMT1^−/−^ cells were utilized. DNA methylation was examined using 5mC fluorescence staining and a 5mC ELISA (Fig. 1). Qualitative differences between WT and DNMT1^−/−^ cells were identified within the nuclei, showing various 5mC-expressing foci in the WT cells (Fig. 1a; white arrows). When focusing on the overall fluorescence intensity of the entire cell nucleus, no significant difference in gray value intensity (GVI) per cell was detected (p = 0.914) (Fig. 1b). However, analysis of the subnuclear localization patterns showed a significant increase of 5mC-foci within the cell nuclei of WT cells when compared with DNMT1^−/−^ cells (p < 0.05) (Fig. 1c). 5mC ELISA analysis exhibited a statistically significant 0.6% decrease in global DNA methylation in the DNMT1^−/−^ cells when compared to WT cells (Fig. 1d; p < 0.05). In the WT cells, 1.4% of all cytosines were methylated, whereas the DNMT1^−/−^ cells contained only 0.8% methylated cytosines (Fig. 1d).

### Raman analysis of WT and DNMT1^−/−^ cells shows differences in nucleic acid bands

**Figure 2:**
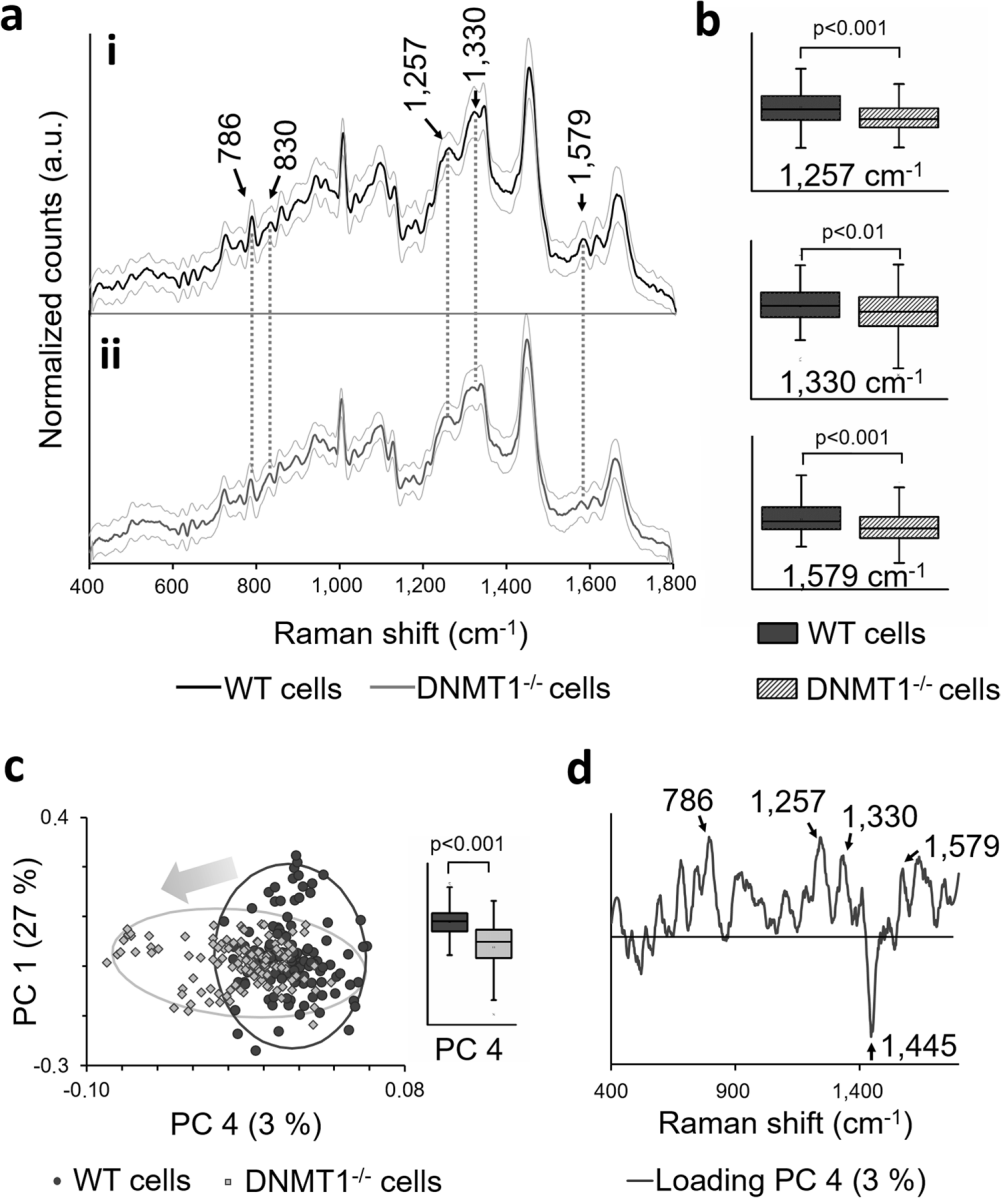
Identification of DNA methylation Raman signatures in WT and DNMT1^−/−^ cells. (**a**) Raman spectra of (i) HCT116 WT and (ii) DNMT1^−/−^ cells. Raman signatures that can be assigned to DNA bands are indicated by arrows. Standard deviations are indicated by the gray lines. n = 125. (**b**) Statistical analysis of significantly increased Raman bands, which can be assigned to DNA methylation. (**c**) Score plot of PC 1 and PC 4 shows a grouping tendency between WT and DNMT1^−/−^ cells illustrated by a gray arrow. The score mean value of both groups for PC 4 differs significantly from each other. Two-tailed *t*-test, n = 125 (**d**) PC 4 Loading describes the Raman shifts that vary in WT cells compared to DNMT1^−/−^ cells. Noticeable DNA peaks are indicated by arrows.

The Raman spectral signature of the HCT116 WT cells displayed a cellular peak pattern consisting of complex overlapping signals from the biochemical cellular components (Fig. 2a,i). DNMT1^−/−^ cells revealed an increased relative Raman signal at 830 cm^−1^ and decreased relative spectral intensities at the Raman bands 786 cm^−1^, 1257 cm^−1^, 1330 cm^−1^, 1342 cm^−1^, 1579 cm^−1^, 1610 cm^−1^ and 1662 cm^−1^ (Fig. 2a,ii). Statistical analysis showed a significant change in intensity at 1257 cm^−1^ (p < 0.001), 1330 cm^−1^ (p < 0.01) and 1579 cm^−1^ (p < 0.001) (Fig. 2b).

For further analysis of the spectral data, PCA was performed. PCA is commonly used to resolve complex spectral peak shifts and to reveal spectroscopic variations and peak correlations by calculating principal components (PCs), which dissolve the variances within the spectra data set^32,33^. In the score plot (Fig. 2c), every data point represents a Raman spectrum of a single cell. The score values were used to validate significant differences in the Raman spectra of WT cells and DNMT1^−/−^ cells. In the PC 4 scores plot, which describes 3% of the total spectral variances, WT cells and DNMT1^−/−^ cells showed a separation tendency. Statistical analysis revealed significant differences of the PC 4 score value between WT and DNMT1^−/−^ cells (p < 0.001) (Fig. 2c). Other PC scores did not show significant differences (Suppl. Fig. 3). In general, loadings of each PC demonstrate the spectral changes, which determine the position of the PC score values^33^. Positive peaks in the loadings indicate increased Raman signals in the original spectra of the positive score values. In contrast, negative loading values represent increased Raman signals in the spectra of the negative score values^33^. Based on the PC 4 loading, increased bands at 786 cm^−1^, 1257 cm^−1^, 1330 cm^−1^ and 1579 cm^−1^ were identified in the spectra of WT cells (Fig. 2d, arrows). DNMT1^−/−^ cells showed an increased Raman signal at 1445 cm^−1^. However, the loading was quite noisy, which indicates that only slight differences of the Raman signals characterize the PC 4. Loadings of PC 1, 2 and 3 showed mostly the spectral baseline (Suppl. Fig. 3).

### 2i medium-adapted mESCs retain their pluripotency and show a significant global demethylation of genomic DNA

In order to reduce global DNA methylation, mESCs were adapted to serum-free 2i medium (2i mESCs) as previously described^31^. Before DNA methylation analysis, the pluripotency of the control and 2i mESCs was verified via Nanog and Oct4 protein expression by IF staining (Suppl. Fig. 2). As expected, both control and 2i mESCs showed Nanog and Oct4 protein expression (Suppl. Fig. 2a and b); however, semi-quantitative analysis of fluorescence intensities revealed a significantly higher Nanog expression in mESCs cultured in 2i medium when compared with the ESCs that were cultured in serum-containing control medium (Suppl. Fig. 2a). In addition, *Nanog* gene expression of 2i mESCs was 3-fold higher (p < 0.05) compared to the control mESCs, and *Oct4* gene expression of 2i mESCs was 2-fold higher compared to control mESCs (Suppl. Fig. 2c).

**Figure 3:**
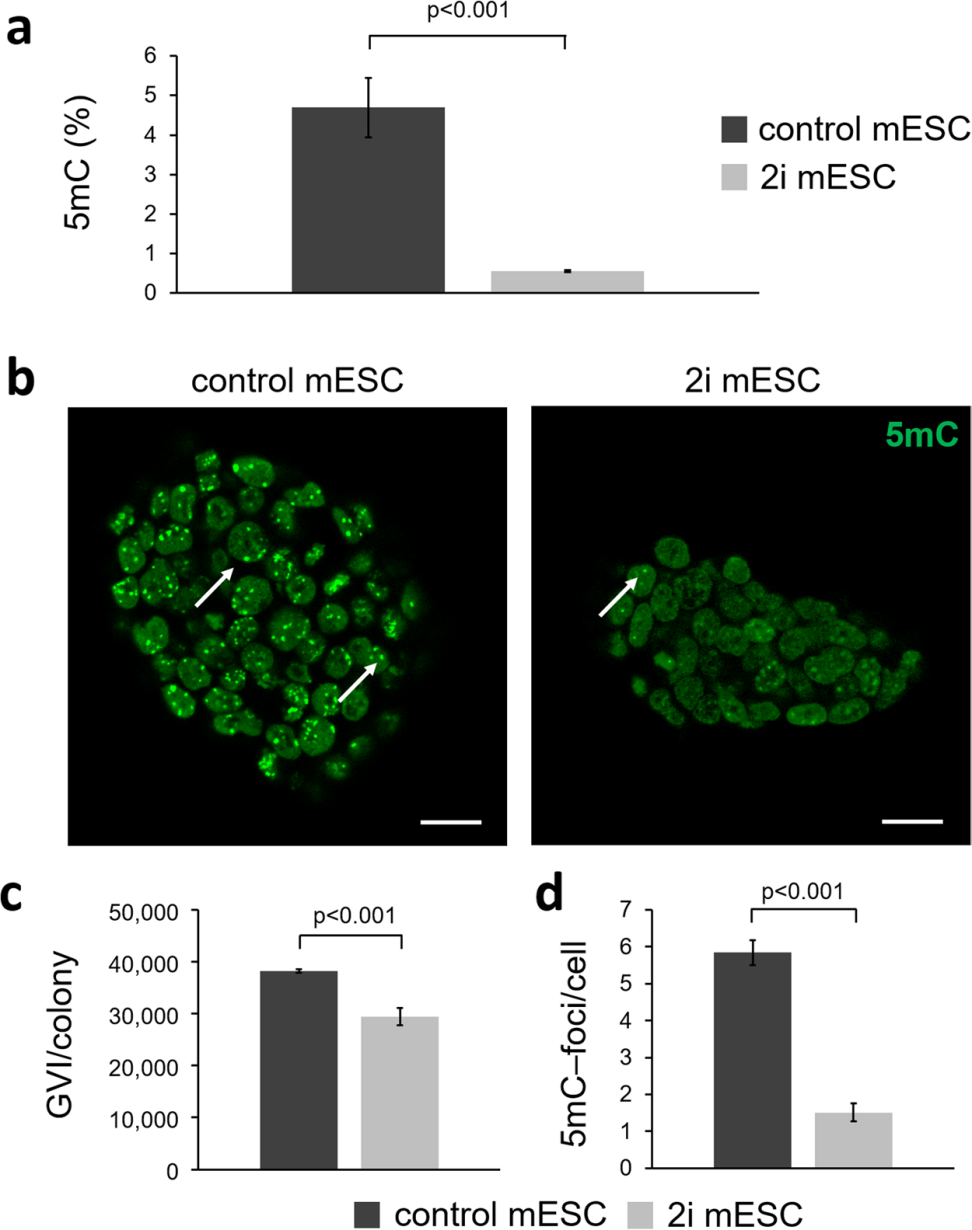
Investigation of global DNA methylation changes. (**a**) 5mC expression in mESCs cultured under control conditions or in 2i medium. 2i medium-adapted cells show a significantly lower DNA methylation. Two-tailed *t*-test, n = 3. (**b**) Immunofluorescence staining of 5mC. Scale bars equal 20 µm. (**c** + **d**) Quantitative fluorescence intensity analysis of 5mC-stained mESCs. 2i medium-cultured mESCs show a significant lower mean intensity and less 5mC-foci/cell (indicated by white arrows in **b**) when compared with cells cultured in the presence of serum (control). Two-tailed *t*-test, n = 3.

mESC adaption to serum-free 2i medium results in a massive global demethylation of the genomic DNA^34,35^. For the determination of global DNA methylation levels in control versus 2i mESCs, a 5mC ELISA was performed revealing a significantly decreased relative cytosine 5mC of 4% (4.7% in control mESCs before adaption and 0.7% in 2i mESCs after adaption) (Fig. 3a; p < 0.001). This result was confirmed by 5mC IF staining (Fig. 3b). The semi-quantitative fluorescence intensity analysis of the whole nucleus exhibited significantly lower GVI per colony in the 2i mESCs when compared with the control mESCs (Fig. 3c). By comparing subnuclear localization patterns of 5mC, we further identified that the control mESCs exhibited significantly more 5mC-foci within each cell nucleus (Fig. 3b, white arrows, and Fig. 3d).

### Raman microspectroscopy revealed altered nucleic acid signals in 2i medium-adapted mESCs

**Figure 4:**
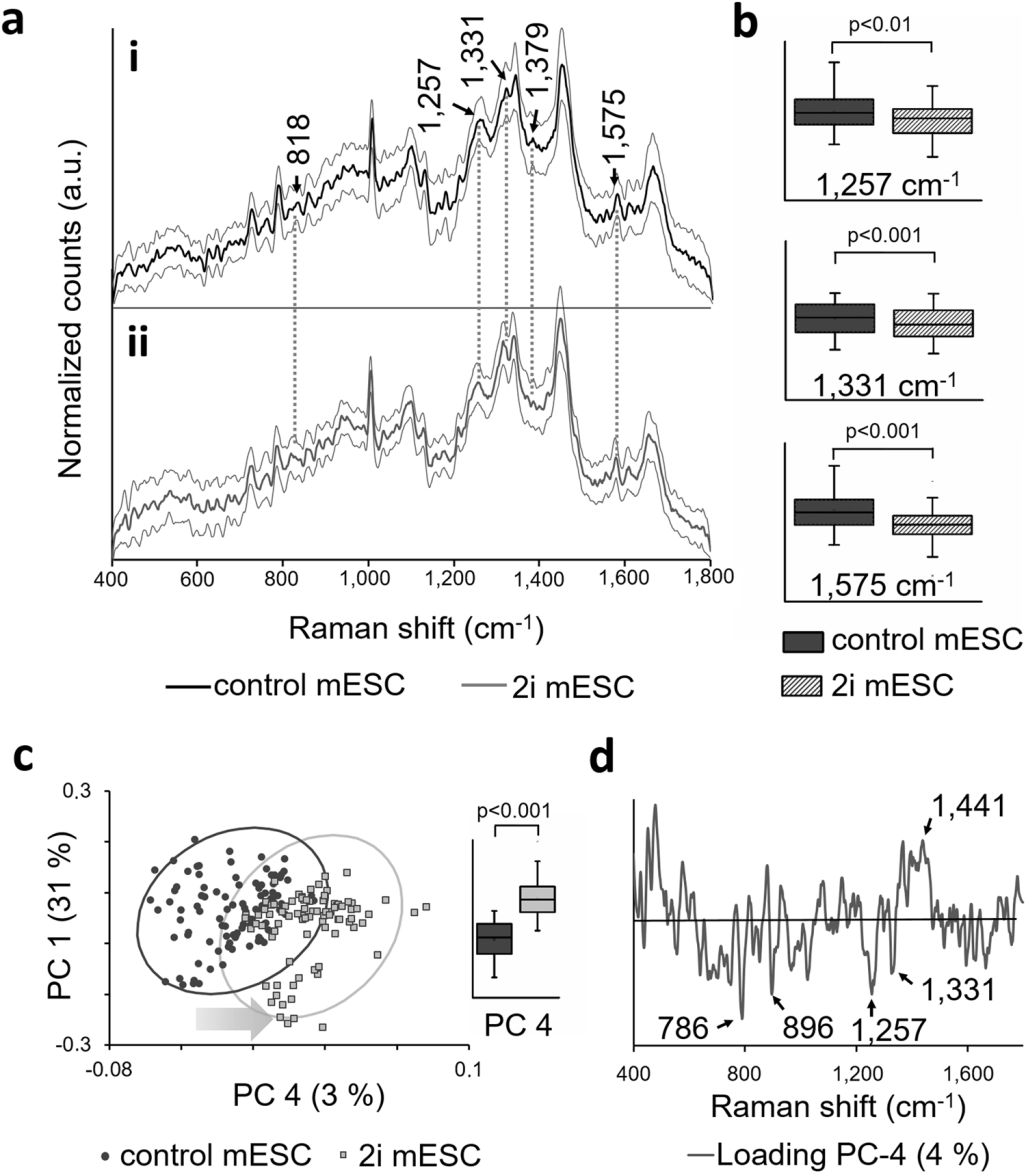
Identification of mESC Raman signatures due to an altered global DNA methylation. (**a**) Raman spectra of mESCs cultured in (i) control and (ii) 2i medium. Raman signatures that can be assigned to DNA bands are indicated by black arrows. The standard deviation is indicated by the gray lines. (**b**) Statistical analysis of significantly increased Raman bands, which can be assigned to DNA methylation. Two-tailed *t*-test, n = 85. (**c**) Score plot of PC 1 and PC 3 shows a grouping tendency of mESCs cultured in control and 2i media. The direction of the gray arrow indicates the loss of DNA methylation. The score mean value of both groups for PC 4 differs significantly from each other. Two-tailed *t*-test, n = 85 (**d**) PC 4 loading describes the Raman shifts that vary in 2i-adapted mESCs compared with mESCs cultured in the control medium. Peaks of interest are indicated by black arrows.

Control mESCs and 2i mESCs were investigated with Raman microspectroscopy. Non-stained control and 2i mESCs showed defined peak patterns containing complex overlapping signals from lipids, proteins, carbohydrates and nucleic acids^36^. Several differences in relative intensities between control mESCs (Fig. 4a,i) and 2i mESCs (Fig. 4a,ii) were identified. The most prominent ones, which can be assigned to nucleic acid bands, are highlighted with black arrows (Fig. 4a). Most prominent increased Raman bands in control mESCs were detected at 1257 cm^−1^, 1331 cm^−1^, 1379 cm^−1^ and 1575 cm^−1^. 2i mESCs exhibited an increasing relative intensity at 818 cm^−1^. Statistical significant decreased Raman shifts were identified at 1257 cm^−1^ (p < 0.01), 1331 cm^−1^ (p < 0.001) and 1575 cm^−1^ (p < 0.001) (Fig. 4b). The corresponding PCA analysis showed separated clusters for control and 2i mESCs in PC 4, which explained 3% of the total spectral variance. The PC 4 score values of control and 2i mESCs differed significantly (p < 0.001) (Fig. 4c). The corresponding loading of PC 4 showed decreased Raman bands (786 cm^−1^, 896 cm^−1^, 1257 cm^−1^, 1331 cm^−1^) and increased signals (481 cm^−1^, 1441 cm^−1^) for 2i mESCs when compared to control mESCs. In general, we noted that the loadings were quite noisy, which indicates that only slight differences of the Raman signals characterize the PC 4 (Fig. 4d). For the other PC scores, no separation tendency was detected. Scores and loadings of PC 1–3 are shown in Suppl. Fig. 4.

### Raman spectra differentiate between low and high methylation status independent of the cell type

**Figure 5:**
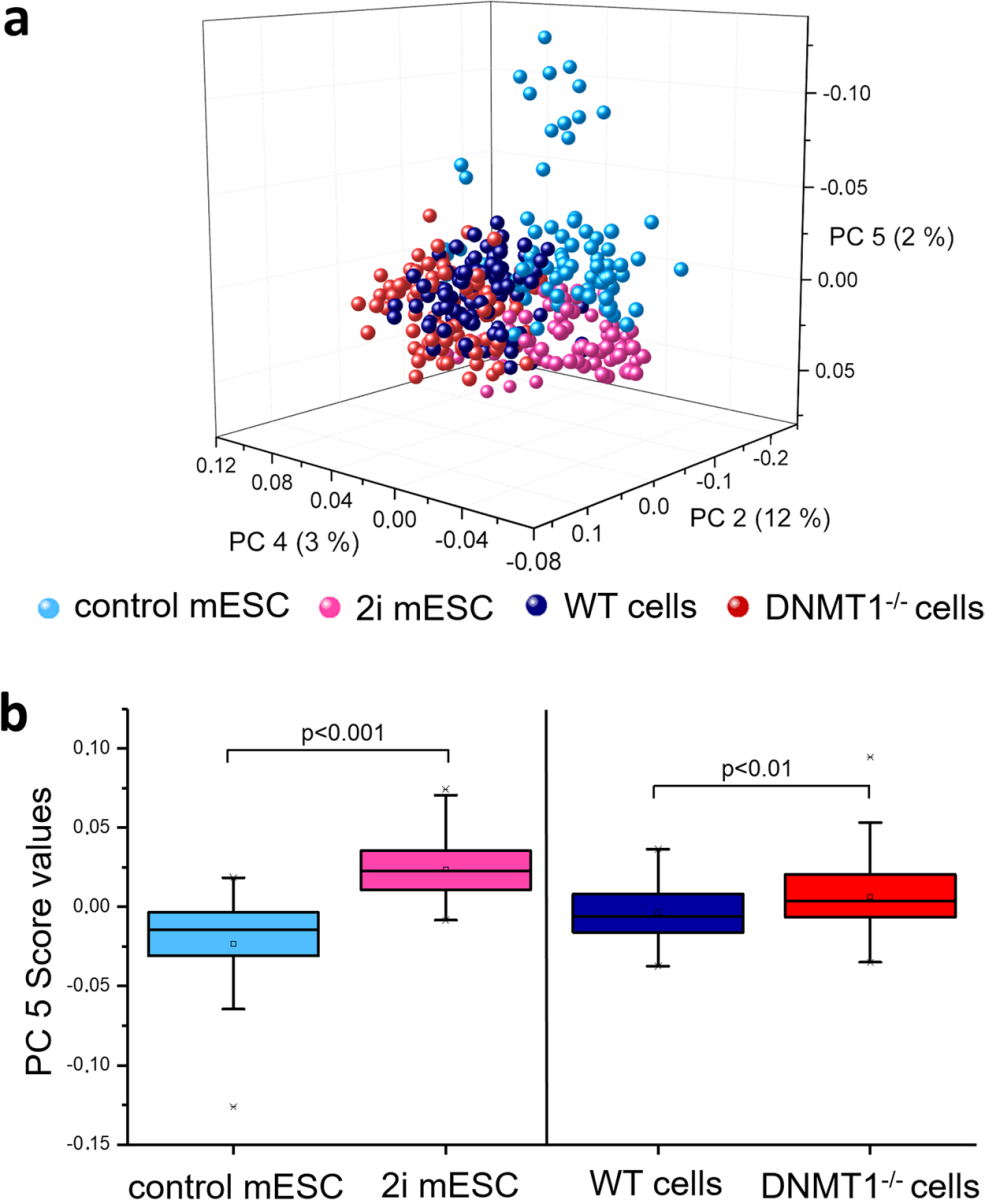
Comparison of Raman spectra from mouse pluripotent stem cells cultured in either control or 2i medium with human colon cancer WT and DNMT^−/−^ cells. (**a**) 3D score plot of Raman spectra from control mESCs, 2i mESCs as well as human colon cancer WT and DNMT1^−/−^ cells showing PC 2, PC 4 and PC 5. There is a grouping tendency between control mESCs and WT cells (blue shades), as well as 2i mESCs, DNMT1^−/−^ cells (green shades). (**b**) The grouping tendency is visualized by a box plot of PC 5 score values. It shows significantly lower scores for control mESCs and WT cells when compared with 2i mESC and DNMT1^−/−^ cells. One-way ANOVA, n = 30.

To further investigate whether the differences in Raman spectra of the low and high global DNA methylation status are independent of species and cell types, PCA were performed on the whole data sets consisting of the spectra from all, the mESCs (control and 2i mESCs) and human colon cancer (HCT116 WT and DNMT1^−/−^) cells. A 3D score plot of PC 2 (12%), PC 4 (3%) and PC 5 (2%) revealed separations between the samples (Fig. 5a). PC 2 reflects differences between cell types and separates between mESC as well as the WT and DNMT1^−/−^ cells. In addition, PC 5 revealed a separation tendency between the high-methylated mESC control and the HCT116 WT cells, and the low-methylated 2i mESC and DNMT1^−/−^ cells. This separation is also illustrated in Fig. 5b, showing the mean score values of PC 5. The score values of the control mESCs differed significantly from the score values of the 2i mESCs (p < 0.001), and the score values of the HCT116 WT cells differed significantly from the score values of the DNMT1^−/−^ (p < 0.01).

The most prominent Raman peaks that revealed altered relative intensities in Raman spectra of the high- and low-methylated mESCs and the high- and low-methylated human colon cancer WT and DNMT1^−/−^ cells are summarized in Table 1. Most of them show increased signals for high level of DNA methylation (786 cm^−1^, 1257 cm^−1^, 1317–1323 cm^−1^, 1335 cm^−1^, 1342 cm^−1^, 1379 cm^−1^, 1579 cm^−1^, 1605–1610 cm^−1^and 1662 cm^−1^). The Raman band at 815–830 cm^−1^ revealed a decreased relative intensity in the high-methylated cells.

**Table 1:** Identified Raman peaks that are altered due to global DNA methylation in mESCs and human colon cancer cells.

Raman Shift (cm^−1^)	Assignment	↑↓ relative Raman intensity for high-methylated cells
786	5-Methylcytosine^52^, Cytosine, Thymine, Phosphate backbone of DNA/RNA^39,47–49^	**↑**
815–830	Phosphate backbone of DNA/RNA^54^	**↓**
1257	Cytosine, Adenine^38,41^	**↑**
1317–1323	Guanine^41,47^	**↑**
1335	CH_2_CH_3_ wagging^37^	**↑**
1342	Guanine^47^	**↑**
1379/1386	CH_3_^39,53^	**↑**
1579	Pyrimidine ring^39^	**↑**
1605–1610	Cytosine^41^	**↑**
1662	DNA^58,59^	**↑**

### Raman imaging identifies 5mC-foci on a subnuclear level

**Figure 6:**
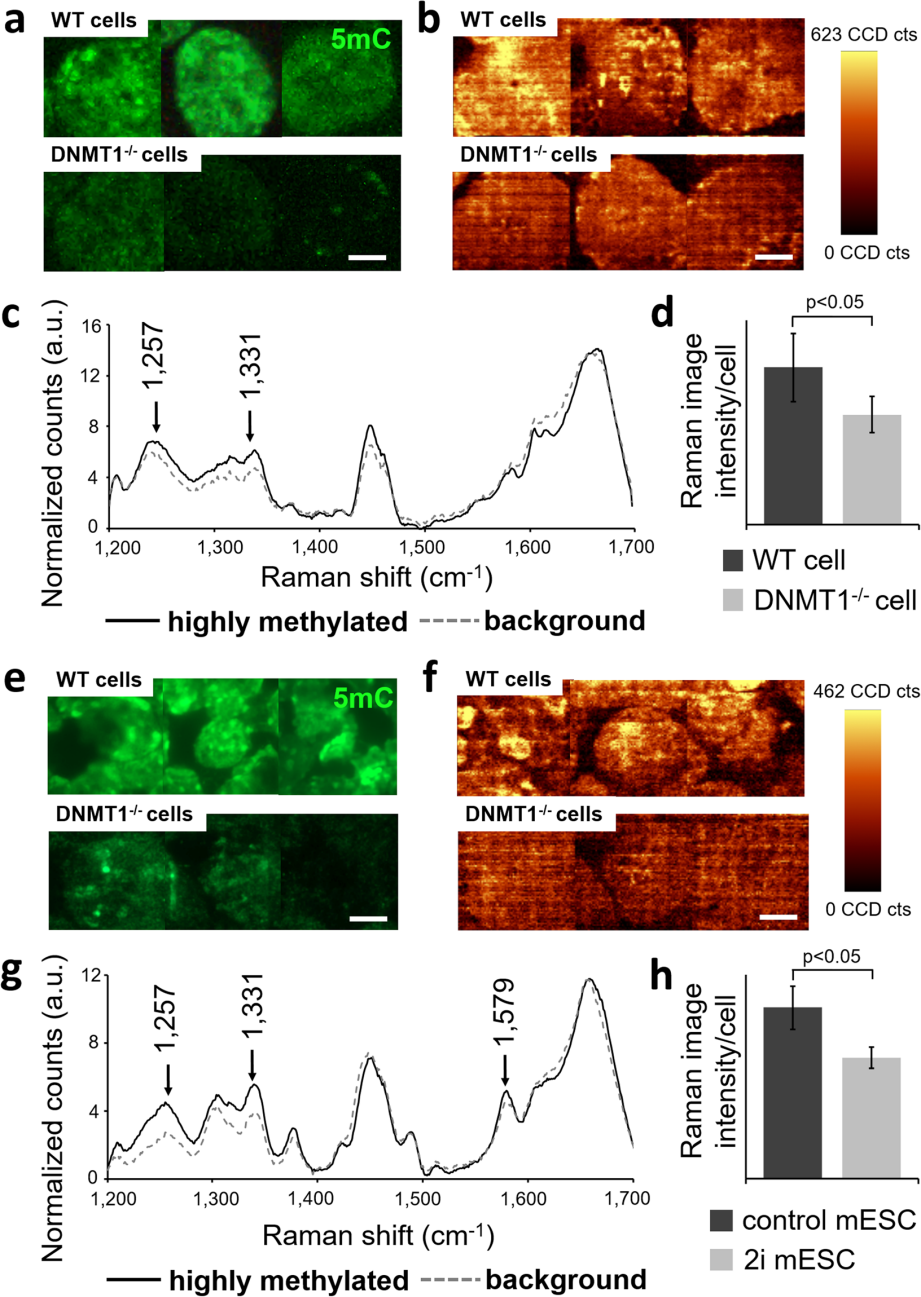
Detection of 5mC-foci with high-resolution Raman imaging of HCT116 cells and mESCs. (**a**) Anti-5mC immunofluorescence staining of HCT116 WT and DNMT1^−/−^ cells. (**b**) Heat maps of the Raman images obtained from HCT116 WT and DNMT1^−/−^ cells using the sum intensities of 1257 cm^−1^, 1379 cm^−1^ and 1579 cm^−1^. (**c**) Average spectra from high-intensity regions and low-intensity regions of the heat maps from panel (b). (**d,h**) Statistical analysis of the Raman images from panels b and f. Two-tailed *t*-test, n = 3. (**e**) Anti-5mC immunofluorescence staining of mESC control and 2i mESCs. (**f**) Heat maps of the Raman images from mESC control and 2i mESCs using the sum intensities described in panel b. (**g**) Average spectra from high-intensity regions and low-intensity regions of the heat maps from panel f. Scale bars in all images equal 5 µm.

Since the differences in the Raman spectra between the investigated groups were rather subtle, high-resolution Raman imaging was additionally performed to obtain laterally-resolved spectral information of the 5mC–foci within the cell nuclei. HCT16 WT and DNMT1^−/−^ cells (Fig. 6a–d) as well as control and 2i mESCs (Fig. 6e–h) were scanned with a resolution of 0.2 × 0.2 µm. A heat map of both cell types was created using the sum of the intensities at 1257 cm^−1^, 1331 cm^−1^ and 1579 cm^−1^. The heat maps of the resulting Raman images showed specific structures within the nucleus that were comparable to the structures seen when performing 5mC IF staining (Fig. 6a,b,e,f). In the average spectra from high-intensity (highly methylated) regions and low-intensity (background) regions of the heat maps, increased Raman peaks in the spectral region of 1250–1390 cm^−1^ were observed for both cell types (Fig. 6c,g). The semi-quantitative pixel intensity analysis of the heat maps exhibited significantly lower values for the HCT116 DNMT1^−/−^ cells and 2i mESCs when compared with the HCT116 WT cells and control mESCs (Fig. 6d,h; p < 0.05).

## Discussion

It this study, we demonstrated that Raman microspectroscopy and Raman imaging have the potential to detect DNA methylation states in living cells. We utilized different cell types from human and mouse origin that differ in their global DNA methylation level, and identified specific Raman signals that can be employed to monitor methylated DNA *in situ*.

In a first step, by measuring 5mC and methylated DNA, we identified specific Raman bands at 1257 cm^−1^, 1335 cm^−1^, 1379 cm^−1^ and 1579 cm^−1^ that can be assigned to nucleobases or CH_3_ (Table 1)^37–41^. These results demonstrate that differences between methylated and non-methylated DNA can be detected by Raman microspectroscopy.

Next, we used WT cells of the human colon cancer cell line HCT116 and their DNMT1-hypomorph progeny showing a lower global DNA methylation. We confirmed the decreased global DNA methylation in DNMT1^−/−^ cells by anti-5mC IF staining and 5mC ELISA. Interestingly, in our study, in both the WT and DNMT1^−/−^ cells, the 5mC ELISA revealed a lower global DNA methylation level when compared to previous studies^30^. Literature determined 4.0% methylated cytosines for WT cells, and our results revealed 1.3%. For DNMT1^−/−^ cells, 3.2% of all cytosines were reported to be methylated^30^. In our study, we quantified 0.7% methylated cytosines. Nevertheless, the relative global DNA methylation difference of 0.6% between the samples is similar to what had been previously reported^30^.

Our Raman microspectroscopy measurements showed differences in the Raman signatures of WT and DNMT1^−/−^ cells. PCA revealed a grouping tendency for PC 4 (Fig. 2). Although some overlapping of the populations was seen that might be due to biological variances within the samples, the mean score values revealed a significant difference between the two samples. Most differences in Raman spectra as well as striking peaks in the loading plot could be assigned to nucleic acid bands (Table 1).

The second cell type in this study were mESCs that were either maintained in routine serum-containing control medium, or they were adapted to serum-free 2i medium. As expected, exposure to 2i medium led to massive global DNA demethylation as described before^31,34,35^, which was successfully confirmed by anti-5mC IF staining and 5mC ELISA. Accordingly, the control mESCs revealed a global DNA methylation of 4%^34,35^, and only 1% of all cytosines were methylated in the 2i medium-adapted mESCs^34^. We confirmed that the mESCs maintained their pluripotency after 2i adaption by gene and protein expression profiling focusing on the pluripotency markers Nanog and Oct4, which were increased after two weeks of 2i medium adaption. This observation is conform with a report by Ying *et al*. who postulated that 2i ESCs represent the ground state of pluripotency^31^. Therefore, we hypothesize that the 2i mESCs used in our study were actually closer to the “naïve pluripotent ground state” than mESCs cultured in standard serum-containing medium^31,42^. However, the adaption process of mESCs to serum-free 2i medium seems to be not homogenous and may require modified culture protocols. The gene expression of the pluripotency markers *Nanog* and *Oct4* (Suppl. Fig. 2) as well as the 5mC fluorescence signal (Fig. 3) revealed high variances within the 2i mESC population. Interestingly, Abranches *et al*. made similar observations showing higher Nanog fluctuations in cells that were exposed to 2i medium^43^. One explanation for this could be that some of the cells in our population were already differentiated prior exposure to the 2i medium. Tamm *et al*. examined the capacity of 2i medium to rescue partially-differentiated mESCs by culturing spontaneously-differentiated cells in 2i medium^44^. They found that the cells were not able to return to the ground state anymore^44^.

Employing Raman microspectroscopy, we were able to distinguish between 2i medium-adapted and control mESCs. Overlapping populations in PC 4 can be explained by biological variances within the samples due to the heterogeneity of the 2i mESC population as described above. The most prominent Raman bands, which revealed the major differences, were assigned to nucleic acid bands and methyl groups (Table 1). DNA methylation and remodeling of chromatin are the most striking changes during early embryogenesis^8–10,45^. It can therefore be assumed that the DNA methylation level represents a potential biomarker that can be tracked during early embryonic development. Nevertheless, it must be taken into account that the 2i medium-treated mESCs used in our model system may also show other epigenetic changes, e.g. a reduction of H3K27me3^46^.

In all experiments of this study, we detected altered relative peak intensities in the Raman spectra of cells with different DNA methylation levels. Most of the Raman shifts were assignable to DNA components, nucleobases, phosphate and ribose. We identified marker peaks, which can be assigned to 5mC, such as the Raman shift at 786 cm^−1^. This peak is also associated with cytosine, thymine or phosphate^41,47–51^. However, Barhoumi *et al*. described the appearance of a peak in case of 5mC at the same position^52^. The comparison of the loadings from both cell types in our study revealed a higher relative intensity at 786 cm^−1^ for the cells with a higher DNA methylation (human colon cancer WT cells and control mESCs). A further peak at 1330–1331 cm^−1^, which can be assigned to CH_3_ and CH_3_CH_2_ twisting and wagging modes in nucleic acids, was observed for all highly methylated cells as well as for 5mC and methylated DNA^39,53,54^. In addition, an increased peak between the wavenumbers 1379 cm^−1^ and 1386 cm^−1^, also shown in the Raman spectra of methylated DNA, was detected in the spectra of control mESCs. In this region, previous studies described symmetric CH_3_ bending^39,53^.

In addition to 5mC markers, we observed changes in DNA signals which might be an indirect effect of 5mC. Most predominant peaks that cannot directly be assigned to 5mC but to other nucleobases and potential chromatin changes were observed at 1257 cm^−1^ (thymine, cytosine, adenine)^41,48,49,55^, 1304 cm^−1 ^^28,39^ (cytosine, adenine)^56^, 1323 cm^−1^ (guanine)^57^, 1579 cm^−1^ (pyrimidine ring)^39^ and 1662 cm^−1^ (amide I, nucleic acid modes)^58,59^. These peaks were also detected in the loadings of the corresponding PCs for the compared cell types. In addition, the Raman peaks at 1257 cm^−1^ and 1579 cm^−1^ were also observed in the spectra of methylated DNA. It is still unclear why methylated DNA seems to enhance nucleobase-assigned Raman shifts. A possible explanation for the enhanced Raman shift of thymine could be that it is, similar to cytosine, a pyrimidine ring^39,47,60^. Due to the additional methyl group at C5, the 5mC has a very similar chemical structure when compared to the thymine^61^. Raman signals for 5mC might therefore appear at the same positions as those described previously for thymine.

We analyzed the Raman images of the HCT116 cells and mESCs. Using the sum of the intensities at 1257 cm^−1^, 1331 cm^−1^ and 1579 cm^−1^, a heat map of the Raman images from both cell types was generated. We were able to show a significant intensity increase in the heat maps of the mESC control and HCT116 WT cells when compared with the mESC 2i and HCT116 DNMT1^−/−^ cells (Fig. 6d,h). In addition, the heat maps revealed structures within the nuclei that were similar to the 5mC-foci identified by the anti-5mC IF staining (Fig. 6b,f). These regions are likely to be composed of heterochromatin, since the DNA is densely packed here and thus generates a stronger Raman signal^62^. Since most of the DNA methylation-related consequences are genomic imprinting, DNA methylation is more strongly present in heterochromatic regions^63^, suggesting that the structures can be associated with DNA methylation. Based on our results, we hypothesize that the Raman bands at 1257 cm^−1^, 1331 cm^−1^ and 1579 cm^−1^ are potential markers for DNA methylation.

The overall challenge of this study was to assess DNA methylation changes *in situ*, which represent only a small fraction of all the processes that take place at a certain time in a living cell. Not only the epigenome, but also the genome, transcriptome and proteome of a cell is subject to complex dynamic processes^46^. This might be one of the reasons why some of the detected changes were only minor. In order to obtain statistically even more valid data, more cell types could be included in further investigations, and direct methods for the elimination of DNA methylation such as DNMT 1/3a/3b trible KO could be used.

## Conclusion

Our data demonstrate that Raman microspectroscopy and Raman imaging in combination with PCA possess the required fidelity to detect little but significant changes in DNA methylation status on a single cell level. Here, we employed these technologies to track epigenetic changes in mouse pluripotent stem and human colon cancer cells *in situ*. We also showed that high-resolution Raman imaging can resolve structures on a nano- and microscale within the nucleus of living cells enabling the monitoring of epigenetic processes. Raman microspectroscopy and Raman imaging may ultimately allow scientists to further decipher crucial connections between epigenetics and early human embryogenesis, aging or diseases.

## Methods

### Cell culture

Rhee *et al*. previously generated a HCT116 DNMT1 knockout construct in which exons 3, 4, and 5 of human DNMT1 were replaced with a hygromycin resistance gene. The disruption of DNMT1 led to a reduced global DNA methylation^30^. Human colon cancer HCT116 wildtype cells (ATCC^®^ CCL-247^™^) and DNMT1^−/−^ cells were cultured in RPMI medium 1640 (Thermo Fisher Scientific, Waltham, USA) with 10% FBS and 1% P/S.

CCE mESCs (ATCC^®^ SCRC-1023^™^) were cultured in KO-DMEM (Thermo Fisher Scientific), supplemented with 15% fetal bovine serum, 1% Penicillin-Streptomycin, 1% MEM non-essential amino acids, 1% L-Glutamine, 0.2% HEPES, 0.1% 2-Mercaptoethanol (all Thermo Fisher Scientific) and 0.1% Leukemia inhibitory factor (Merck, Darmstadt, Germany). Cells were split every second day. To obtain naïve mESCs, the cells were adapted to a serum-free culture condition using ESGRO^®^-2i Medium (Merck) according to the manufacturer’s protocol. The adaption took 14 days. Every second day, cells were split.

### Immunofluorescence (IF) staining

IF staining was performed using a 5mC mouse monoclonal IgG antibody (Merck, MABE146) according to a previously described protocol^64^ with slight modifications. Cells were washed with PBS and fixed with 0.25% paraformaldehyde (Sigma-Aldrich, St. Louis, USA) in PBS^−^ for 10 minutes at 37 °C, and 88% methanol at −20 °C for 30 minutes. After washing, the cells were treated with 1 M HCl at 30 °C for 30 minutes and were neutralized with 0.1 M sodium borate (pH 8.5). In order to reduce nonspecific binding, the cells were incubated with 2% goat block solution for 20 minutes at 37 °C. Afterwards, the cells were incubated with the 5mC antibody (1:2000, stock 2 mg/ml) overnight at 4 °C. Finally, the cells were stained with goat anti-mouse IgG Alexa Fluor 488 (1:250, Thermo Fisher Scientific) for 30 minutes at room temperature in the dark. IF staining against Nanog and Oct4 (also known as POU5F1) was done as previously described^65^. As primary antibodies served Oct4 rabbit polyclonal IgG (1:200; ab19857, abcam, Cambridge, UK) and Nanog rabbit polyclonal IgG (1:100, NB100–588, Novus Biologicals, Littleton, USA). Goat anti-rabbit IgG-Alexa Fluor 488 (1:250, Thermo Fisher Scientific) was used as secondary antibody. DAPI (1:1, in DPBS, Roche Diagnostics, Mannheim, Germany) was used for nuclear staining. Imaging was done with an LSM 710 confocal microscope (Carl Zeiss AG, Oberkochen, Germany). ImageJ (NIH) was used for semi-quantitative fluorescence analyses.

### Detection of global DNA methylation using 5mC ELISA

DNA was isolated using the FlexiGene^®^ DNA Kit (Qiagen, Hilden, Germany) as instructed by the manufacturer. The 5mC DNA ELISA Kit (D5325, ZymoResearch, Irvine, USA) was used to quantify global DNA methylation. It is based on the colorimetric detection of 5mC using an anti-5mC antibody. The procedure was done according to the manufacturer’s protocol. The wells were loaded with 100 ng DNA. Absorbance was detected at 405 nm using an Infinite® 200 Pro microplate reader (Tecan Group AG, Männedorf, Switzerland). Levels of 5mC were calculated as the percentage of methylated cytosines in total DNA content based on a standard curve generated using the kit controls.

### Quantitative reverse transcription polymerase chain reaction (RT-qPCR)

RNA was extracted from control and 2i mESC as well as primary-isolated mouse embryonic fibroblasts using RNeasy^®^ Plus Mini Kit (Qiagen) following manufacturer’s protocol and stored at −80 °C prior further use. The amount and purity of the RNA were determined by NanoDrop 2000 spectral photometer analysis (Thermo Scientific). A total amount of 1 µg RNA was used to synthesize cDNA utilizing a transcriptor first strand cDNA synthesis kit (Roche). RT-qPCR was carried out using a QuantiTect SYBR Green PCR kit for mESCs (Qiagen). cDNA was used in qPCR reactions with *Nanog* and *Oct4* primers (Qiagen). The samples were normalized to the housekeeping gene *Gapdh* (Qiagen). All procedures were performed as instructed by the manufacturer. For the quantification of the qPCR data, the ΔΔCt was used to determine fold change in RNA expression patterns.

### Raman microspectroscopy of cytidine, 5mC, methylated and non-methylated DNA standard

Cytidine, 5mC (Sigma Aldrich, St. Louis, USA) as well as methylated and non-methylated DNA standards (D5325-5-1, D5325-5-2, ZymoResearch, Irvine, USA) were measured with a confocal Raman microspectroscope (alpha300R, WiTEC GmbH, Ulm, Germany). Raman spectra were acquired with a 50x air objective (N.A. 0.55; Carl Zeiss GmbH, Jena, Germany), using an excitation wavelength of 532 nm, a laser power of 60 mW and an acquisition time of one second.

### Raman microspectroscopy of single living cells

A custom-built Raman microscope as previously described^65–67^ was employed for measuring single living cells. The device consists of a near-infrared 784 nm diode laser with a maximum output power of 85 mW, which was integrated into a standard fluorescence microscope (IX71, Olympus, Japan). A 60x water immersion objective (Olympus®) with a 1.2 numerical aperture (NA) was used for the Raman measurements, resulting in a laser spot in approximately 1 µm diameter^68^. Spectra were detected using an air-cooled charge couple device camera (Andor, Belfast, UK). All cells were trypsinized using 0.25% Trypsin-EDTA (Thermo Fisher Scientific), centrifuged and resuspended in PBS, and transferred to a glas bottom petri dish (Ibidi, Martinsried, Germany). For each sample, 90 single living cells were measured using 10 accumulations, each 10 seconds. The laser was focused on the center of each cell due to an optical trapping effect^69^. The laser focus targets automatically the densest organelle of each cell, which was previously shown to be the cell nucleus^70^. A reference spectrum of the background (glass bottom petri dish covered with PBS) was taken after every ten cells.

### Analysis of Raman spectra

Details on the pre-processing steps were published previously in detail^65,68^. Pre-treatment of the Raman spectra (background subtraction and baseline correction) was performed using the OPUS 4.2 software (Bruker, Ettlingen, Germany). The spectra were cut from the range of 0–1,935 cm^−1^ to 400–1,800 cm^−1^. In order to perform a PCA, the pre-treated data was imported to the Unscrambler X 14.0 software (Camo Software, Oslo, Norway). Vector normalization (normalized to length 1) was applied on the spectra. Afterwards, a PCA analysis with up to 7 principal components (PCs) was performed. The PCA results were presented in a scores plot and loading plots. To compare cells with high versus low methylation levels, confidence ellipses were calculated using the software Origin Pro 9.1 (OriginLab, Northampton, USA).

### Raman imaging of mESCs and HCT116 cells

For Raman imaging of cells, the confocal Raman microspectroscope alpha300R (WITec GmbH, Ulm, Germany) was employed. mESCs and HCT116 cells were trypsinized using 0.25% Trypsin-EDTA (Thermo Fisher Scientific), centrifuged and resuspended in PBS. Cells were immobilized on a glass slide using the Shandon Cytospin 3 (Thermo Fisher Scientific). The cytospots were dried for 20 min and stored at −20 °C. Prior to Raman imaging, the cells were IF-stained using an antibody against 5mC as described above. Raman images were acquired with a 63x Apochromat water dipping objective (N.A. 1.0; Carl Zeiss GmbH, Jena, Germany). Cells were located and an area of 15 × 15 µm was imaged using a step size of 0.2 µm. A green laser with an excitation wavelength of 532 nm was used. The laser power was set on 60 mW and the acquisition time was 0.5 seconds. mESCs obtained from the control and 2i cultures and HTC WT and DNMT1^−/−^ cells were imaged and compared. Raman images were analyzed using the Project Five 5.1 Plus software (WITec GmbH). A cosmic ray removal and a baseline correction was employed prior to analysis. In order to generate a heat map of selected Raman signals, intensity sum filters for the respective Raman bands were created and summed up. Semi-quantitative analysis of the heat maps was performed with ImageJ.

### Statistical analysis

Except stated otherwise, data are shown in mean ± standard deviation (SD). One-way analysis of variance (ANOVA) was performed to compare data groups. Student’s t-test was performed to compare between two data groups using OriginPro (OriginLab®). Probability values of 95%, 99% and 99.9% (p < 0.05, 0.01, 0.001) were used to determine significance.

## Supplementary information

Supplemental Data

## References

[1] Zhou Y, Kim J, Yuan X, Braun T (2011). Epigenetic modifications of stem cells: A paradigm for the control of cardiac progenitor cells. Circ. Res..

[2] Cheng X, Roberts RJ (2001). AdoMet-dependent methylation, DNA methyltransferases and base flipping. Nucleic Acids Res..

[3] Jurkowska RZ, Jurkowski TP, Jeltsch A (2011). Structure and Function of Mammalian DNA Methyltransferases. Chem Bio Chem.

[4] Knippers, R. *Molekulare Genetik: 68 Tabellen*. (Thieme, 2006).

[5] Sen GL, Reuter JA, Webster DE, Zhu L, Khavari PA (2010). DNMT1 maintains progenitor function in self-renewing somatic tissue. Nature.

[6] Ravichandran M, Jurkowska RZ, Jurkowski TP (2018). Target specificity of mammalian DNA methylation and demethylation machinery. Org. Biomol. Chem..

[7] Sadikovic B, Al-Romaih K, Squire JA, Zielenska M (2008). Cause and consequences of genetic and epigenetic alterations in human cancer. Curr. Genomics.

[8] Geiman TM, Muegge K (2010). DNA methylation in early development. Mol. Reprod. Dev..

[9] Kaneda M (2004). Essential role for de novo DNA methyltransferase Dnmt3a in paternal and maternal imprinting. Nature.

[10] Ito S (2010). Role of Tet proteins in 5mC to 5hmC conversion, ES-cell self-renewal and inner cell mass specification. Nature.

[11] Meissner A (2008). Genome-scale DNA methylation maps of pluripotent and differentiated cells. Nature.

[12] Deng J (2009). Targeted bisulfite sequencing reveals changes in DNA methylation associated with nuclear reprogramming. Nat Biotechnol.

[13] Biniszkiewicz D (2002). Dnmt1 overexpression causes genomic hypermethylation, loss of imprinting, and embryonic lethality. Mol. Cell. Biol..

[14] Hon GC (2012). Global DNA hypomethylation coupled to repressive chromatin domain formation and gene silencing in breast cancer. Genome Res..

[15] Ehrlich M (2009). DNA hypomethylation in cancer cells. Epigenomics.

[16] Toraño EG, Petrus S, Fernandez AF, Fraga MF (2012). Global DNA hypomethylation in cancer: Review of validated methods and clinical significance. Clin. Chem. Lab. Med..

[17] Egger G, Liang G, Aparicio A, Jones PA (2004). Epigenetics in human disease and prospects for epigenetic therapy. Nature.

[18] Saunderson EA (2017). Hit-and-run epigenetic editing prevents senescence entry in primary breast cells from healthy donors. Nat. Commun..

[19] Paz MF (2002). Germ-line variants in methyl-group metabolism genes and susceptibility to DNA methylation in normal tissues and human primary tumors. Cancer Res..

[20] Barton SC (2001). Genome-wide methylation patterns in normal and uniparental early mouse embryos. Hum. Mol. Genet..

[21] Liu, J., Hesson, L. B. & Ward, R. L. Liquid Chromatography Tandem Mass Spectrometry for the Measurement of Global DNA Methylation and Hydroxymethylation. *J. Proteomics Bioinform*. **01** (2013).

[22] Frommer M (1992). A genomic sequencing protocol that yields a positive display of 5-methylcytosine residues in individual DNA strands. Proc. Natl. Acad. Sci. USA.

[23] Brauchle E, Schenke-Layland K (2013). Raman spectroscopy in biomedicine - non-invasive *in vitro* analysis of cells and extracellular matrix components in tissues. Biotechnol. J..

[24] Tipping WJ, Lee M, Serrels A, Brunton VG, Hulme AN (2016). Stimulated Raman scattering microscopy: an emerging tool for drug discovery. Chem. Soc. Rev..

[25] Hehl G (2012). Coherent Raman Scattering Microscopy: New Quantitative and Non-Invasive Tools for Biomedical Research. Biophys. J..

[26] Downes A, Elfick A (2010). Raman spectroscopy and related techniques in biomedicine. Sensors.

[27] Barhoumi A, Zhang D, Tam F, Halas NJ (2008). Surface-enhanced raman spectroscopy of DNA. J. Am. Chem. Soc..

[28] Camafeita LE, Sánchez-Cortés S, García-Ramos JV (1996). SERS of guanine and its alkyl derivatives on gold sols. J. Raman Spectrosc..

[29] Guerrini L, Krpetić Ž, Van Lierop D, Alvarez-Puebla RA, Graham D (2015). Direct surface-enhanced Raman scattering analysis of DNA duplexes. Angew. Chemie - Int. Ed..

[30] Rhee I (2002). DNMT1 and DNMT3b cooperate to silence genes in human cancer cells. Nature.

[31] Ying Q-L (2008). The ground state of embryonic stem cell self-renewal. Nature.

[32] Bonnier F, Byrne HJ (2012). Understanding the molecular information contained in principal component analysis of vibrational spectra of biological systems. Analyst.

[33] Lavine B (2003). A user-friendly guide to multivariate calibration and classification, Tomas Naes, Tomas Isakson, Tom Fearn & Tony Davies, NIR Publications, Chichester, 2002, ISBN 0-9528666-2-5, £45.00. J. Chemom..

[34] Leitch HG (2013). Naive pluripotency is associated with global DNA hypomethylation. Nat. Struct. Mol. Biol..

[35] Stadler MB (2011). DNA-binding factors shape the mouse methylome at distal regulatory regions. Nature.

[36] Movasaghi Z, Rehman S, Rehman IU (2007). Raman Spectroscopy of Biological Tissues. Appl. Spectrosc. Rev..

[37] Huang Z (2003). Near-infrared Raman spectroscopy for optical diagnosis of lung cancer. Int. J. Cancer.

[38] Chan JW (2006). Micro-Raman Spectroscopy Detects Individual Neoplastic and Normal Hematopoietic Cells. Biophys. J..

[39] Stone N, Kendall C, Smith J, Crow P, Barr H (2004). Raman Spectroscopy for Identification of Epithelial Cancers. Faraday Discuss..

[40] Katainen E (2007). Quantification of the Amphetamine Content in Seized Street Samples by Raman Spectroscopy. J. Forensic Sci..

[41] Ruiz-Chica AJ, Medina MA, Sánchez-Jiménez F, Ramírez FJ (2004). Characterization by Raman spectroscopy of conformational changes on guanine–cytosine and adenine–thymine oligonucleotides induced by aminooxy analogues of spermidine. J. Raman Spectrosc..

[42] Guo G (2010). Resolution of cell fate decisions revealed by single-cell gene expression analysis from zygote to blastocyst. Dev. Cell.

[43] Abranches E (2014). Stochastic NANOG fluctuations allow mouse embryonic stem cells to explore pluripotency. Development.

[44] Tamm C, Pijuan Galitó S, Annerén C (2013). A comparative study of protocols for mouse embryonic stem cell culturing. PLoS One.

[45] Kafri T (1992). Developmental pattern of gene-specific DNA methylation in the mouse embryo and germ line. Genes Dev..

[46] Marks H (2012). The transcriptional and epigenomic foundations of ground state pluripotency. Cell.

[47] Notingher I (2004). Discrimination between ricin and sulphur mustard toxicity *in vitro* using Raman spectroscopy. J. R. Soc. Interface.

[48] Puppels GJ, Garritsen HS, Segers-Nolten GM, de Mul FF, Greve J (1991). Raman microspectroscopic approach to the study of human granulocytes. Biophys. J..

[49] Overman SA (1998). Conformation and interactions of the packaged double-stranded DNA genome of bacteriophage T7. Biospectroscopy.

[50] Notingher I, Hench LL (2006). Raman microspectroscopy: a noninvasive tool for studies of individual living cells *in vitro*. Expert Rev. Med. Devices.

[51] Sathuluri RR, Yoshikawa H, Shimizu E, Saito M, Tamiya E (2011). Gold Nanoparticle-Based Surface-Enhanced Raman Scattering for Noninvasive Molecular Probing of Embryonic Stem Cell Differentiation. PLoS One.

[52] Barhoumi A, Halas NJ (2011). Detecting chemically modified DNA bases using surface-enhanced raman spectroscopy. J. Phys. Chem. Lett..

[53] Katainen E (2007). Quantification of the amphetamine content in seized street samples by Raman spectroscopy. J. Forensic Sci..

[54] Cheng WT, Liu MT, Liu HN, Lin SY (2005). Micro-Raman spectroscopy used to identify and grade human skin pilomatrixoma. Microsc. Res. Tech..

[55] Notingher I (2002). *In situ* characterisation of living cells by Raman spectroscopy. Spectroscopy.

[56] Binoy J (2004). NIR-FT Raman and FT-IR spectral studies and ab initio calculations of the anti-cancer drug combretastatin-A4. J. Raman Spectrosc..

[57] Coluccio ML (2015). From nucleotides to DNA analysis by a SERS substrate of a self similar chain of silver nanospheres. J. Opt..

[58] Mahadevan-Jansen A, Richards-Kortum RR (1996). Raman spectroscopy for the detection of cancers and precancers. J. Biomed. Opt..

[59] Barr H, Dix T, Stone N (1998). Optical spectroscopy for the early diagnosis of gastrointestinal malignancy. Lasers Med. Sci..

[60] Farquharson S, Shende C, Inscore FE, Maksymiuk P, Gift A (2005). Analysis of 5-fluorouracil in saliva using surface-enhanced Raman spectroscopy. J. Raman Spectrosc..

[61] Johnson TB, Coghill RD (1925). Researches on pyrimidines. C111. The discovery of 5-methyl-cytosine in tuberculinic acid, the nucleic acid of the tubercle bacillus. J. Am. Chem. Soc..

[62] Zhang X (2012). Label-free live-cell imaging of nucleic acids using stimulated raman scattering microscopy. Chem Phys Chem.

[63] Mathieu O, Picard G, Tourmente S (2002). Methylation of a euchromatin-heterochromatin transition region in Arabidopsis thaliana chromosome 5 left arm. Chromosome Res..

[64] Milutinovic S, Zhuang Q, Niveleau A, Szyf M (2003). Knockdown of DNA methyltransferase 1 triggers an intra-S-phase arrest of DNA replication and induction of stress response genes. J. Biol. Chem..

[65] Brauchle E (2016). Non-invasive Chamber-Specific Identification of Cardiomyocytes in Differentiating Pluripotent Stem Cells. Stem Cell Reports.

[66] Pudlas M (2011). Raman Spectroscopy: A Noninvasive Analysis Tool for the Discrimination of Human Skin Cells. Tissue Eng. Part C Methods.

[67] Votteler M (2012). Raman spectroscopy for the non-contact and non-destructive monitoring of collagen damage within tissues. J. Biophotonics.

[68] Brauchle E, Thude S, Brucker SY, Schenke-Layland K (2014). Cell Death Stages in Single Apoptotic and Necrotic Cells Monitored by Raman Microspectroscopy. Sci. Rep..

[69] Ashkin A (1970). Acceleration and Trapping of Particles by Radiation Pressure. Phys. Rev. Lett..

[70] Perney NMB, Horak P, Hanley NA, Melvin T (2012). The self-orientation of mammalian cells in optical tweezers—the importance of the nucleus. Phys. Biol..

