# Supplementary material for: Non-invasive detection of DNA methylation states in carcinoma and pluripotent stem cells using Raman microspectroscopy and imaging
Source: Sci Rep. 2019 May 7;9:7014. doi: 10.1038/s41598-019-43520-z (PMC6504883; doi:10.1038/s41598-019-43520-z)
Supplement: Supplementary file 1 — Supplemental Data [file 41598_2019_43520_MOESM1_ESM.docx]

**Non-invasive detection of DNA methylation states in carcinoma and pluripotent stem cells using Raman microspectroscopy and imaging**

Ruben Daum^1,2^, Eva Brauchle^1, 2^, Daniel Alejandro Carvajal Berrio^1^, Tomasz P. Jurkowski^3^ and Katja Schenke-Layland^1, 2, 4^*

^1^ Department of Women’s Health, Research Institute for Women's Health, Eberhard-Karls-University Tübingen, Silcherstr. 7/1, 72076 Tübingen, Germany

^2^ The Natural and Medical Sciences Institute (NMI) at the University of Tübingen, Markwiesenstr. 55, 72770 Reutlingen, Germany

^3^ Department of Biochemistry, Institute of Biochemistry and Technical Biochemistry, University of Stuttgart, Allmandring 31, 70569 Stuttgart, Germany

^4^ Department of Medicine/ Cardiology, Cardiovascular Research Laboratories, David Geffen School of Medicine at UCLA, 675 Charles E. Young Drive South, MRL 3645, Los Angeles, CA, USA

*** Correspondence to Katja Schenke-Layland**:

Department of Women’s Health, Research Institute for Women's Health, Eberhard-Karls-University Tübingen, Silcherstr. 7/1, 72076 Tübingen, Germany. katja.schenke-layland@med.uni-tuebingen.de, Tel: +49 70712985205, www.schenke-layland-lab.de

**Supplemental Figures**


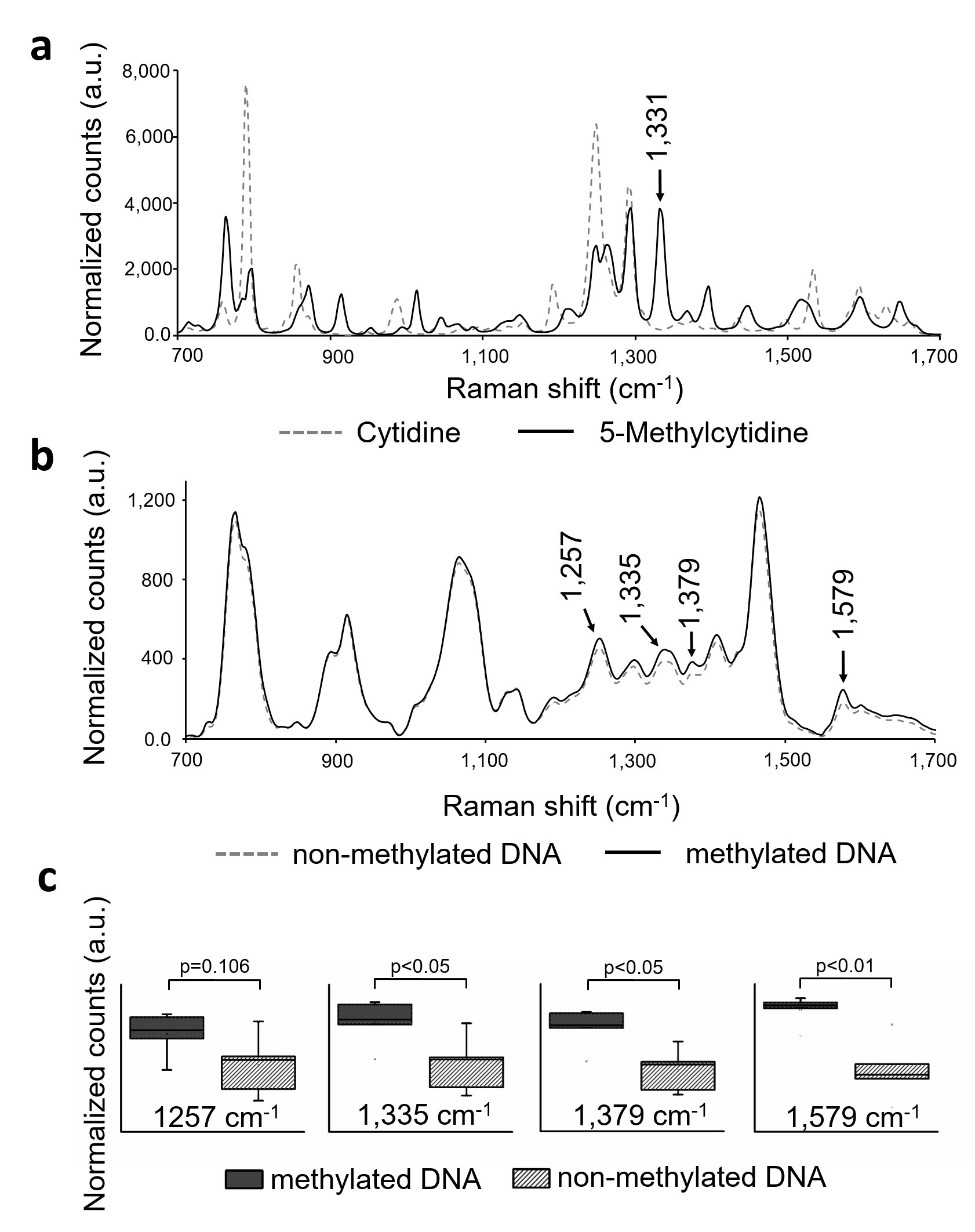


**Suppl. Fig. 1. Raman spectra of cytidine, 5mC, methylated and non-methylated DNA**

(**a**) Raman spectra of cytidine and 5mC show various different intensities with a distinct increased Raman band for 5mC at 1331 cm^-1^. (**b**) Raman spectra of methylated and non-methylated DNA. Increased Raman bands for methylated DNA are indicated by arrows. (**c**) The methylated DNA shows significant increased Raman bands at 1335 cm^-1^, 1379 cm^-1^ and 1579 cm^-1^. Two-tailed *t*-test, n=5.

**
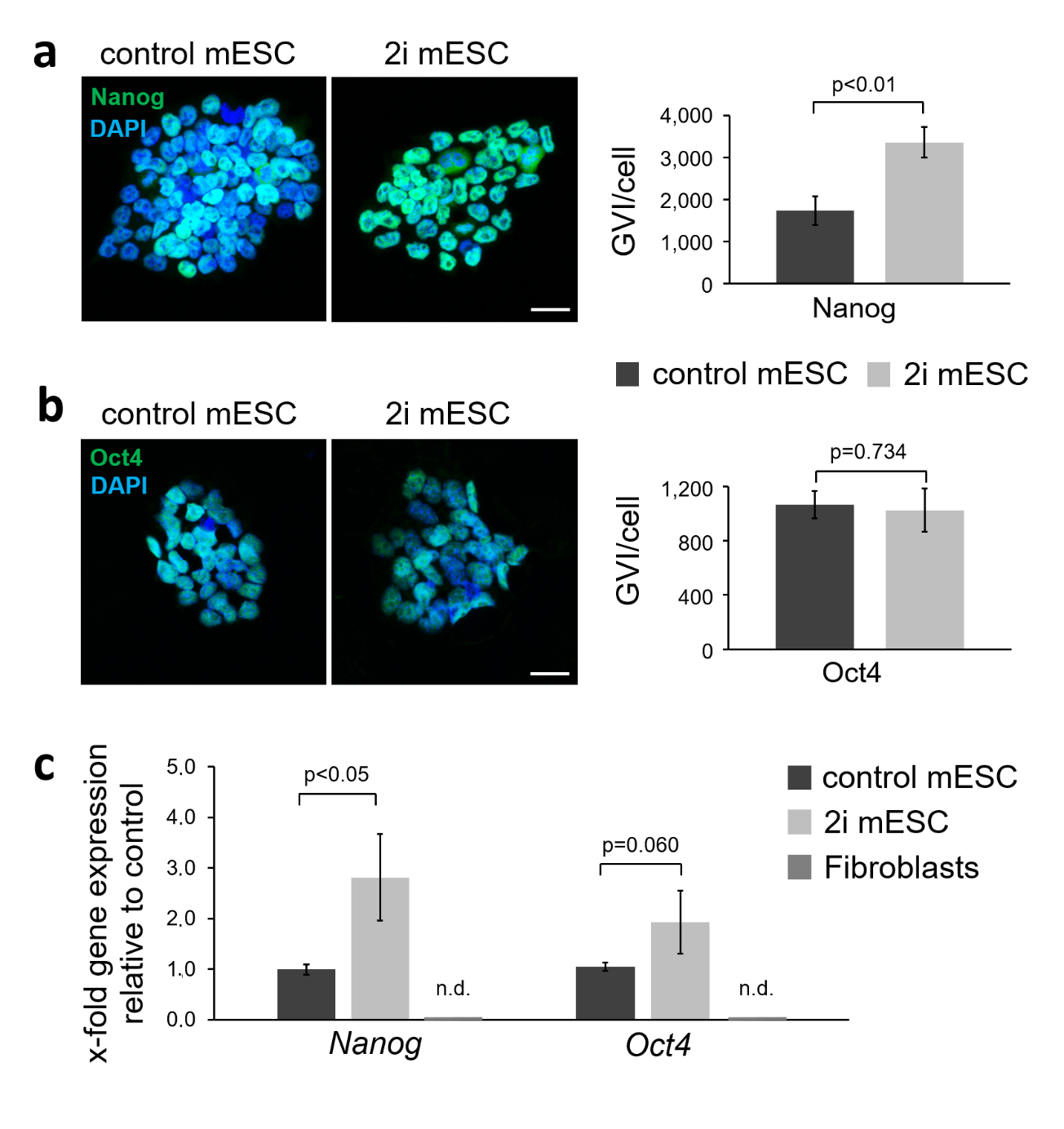
**

**Suppl. Fig. 2. Verification of the pluripotency of mESCs adapted to 2i medium**

(**a**) Nanog and (**b**) Oct4 IF staining (green) of mESCs cells cultured in either control (control mESC) or 2i medium (2i mESC). Nanog staining shows significantly higher GVI/cell values in 2i medium-adapted mESCs. Two-tailed *t*-test, n=3. Scale bars equal 20 µm. (**c**) Relative expression of *Nanog* and *Oct4* in control mESC or 2i mESC. Two-tailed *t*-test, n=3

**
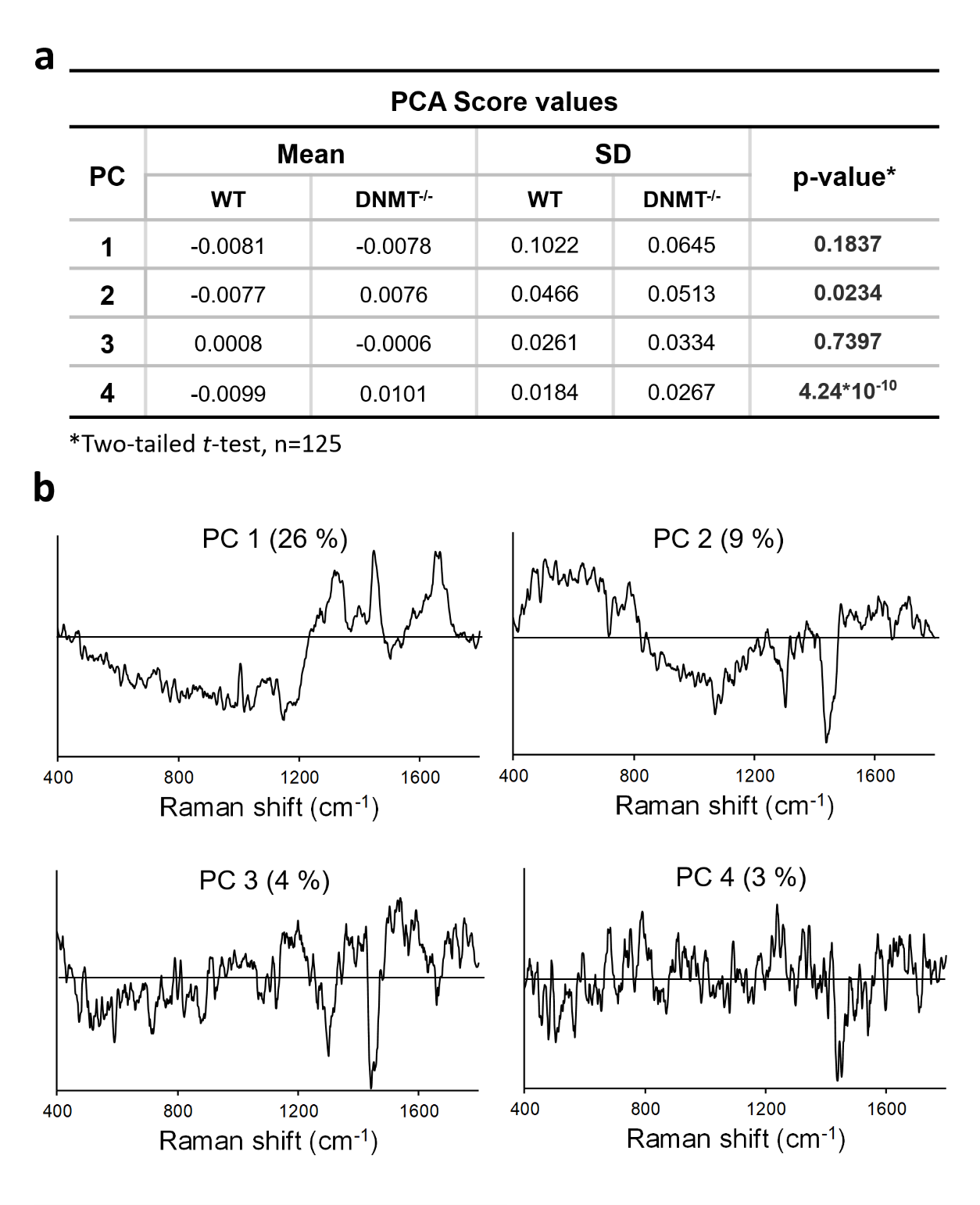
**

**Suppl. Fig. 3. PCA score values and loadings of the human colon cancer cell experiments**

(**a**) Mean values, standard deviations and p-values of the PC score values from WT versus DNMT1^-/-^ cells. Two-tailed *t*-test, n=125. (**b**) PC 1 – PC 3 loadings of the WT versus DNMT1^-/-^ cells.


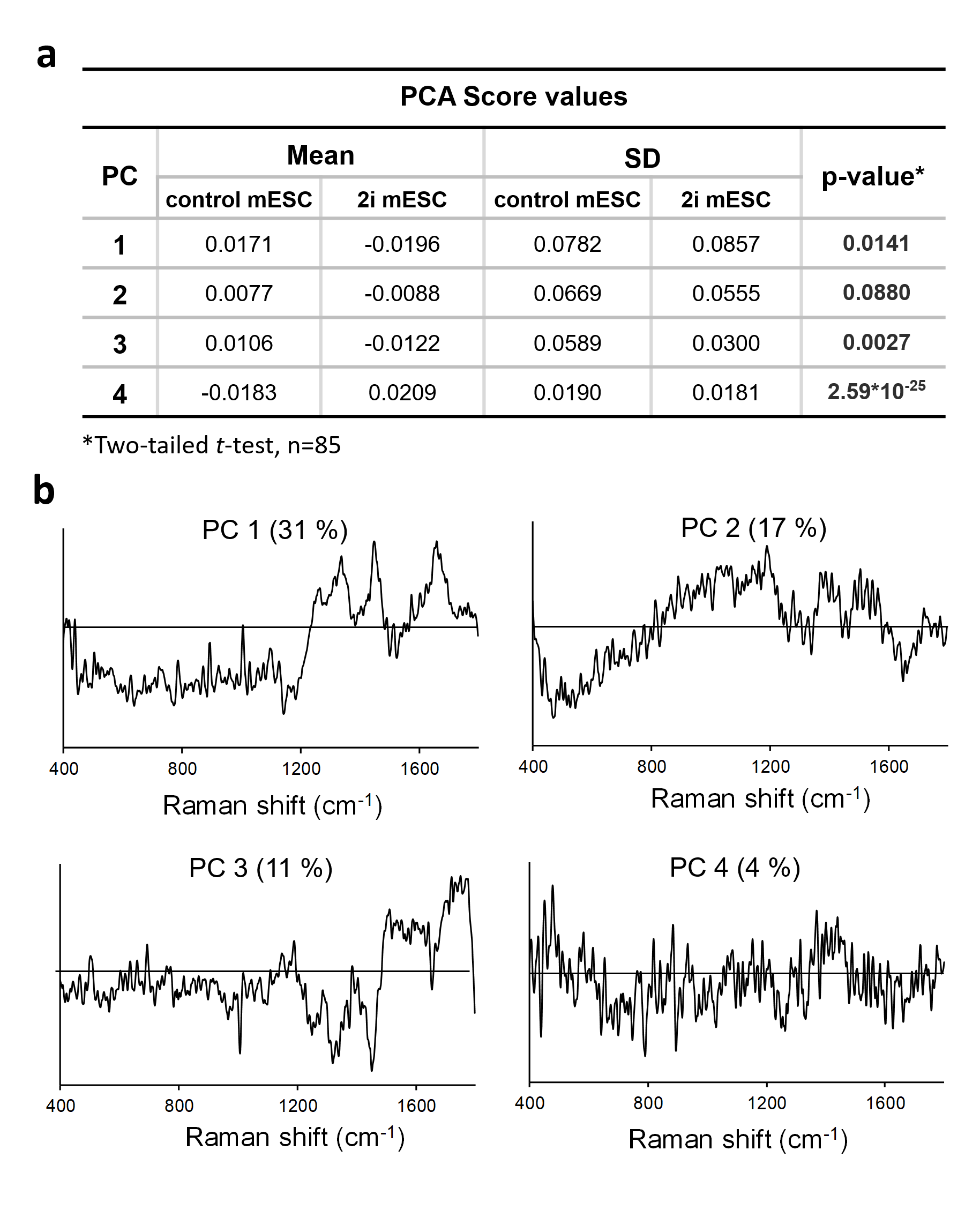


**Suppl. Fig. 4. PCA scores and loadings of the mESC experiments**

(**a**) Mean values, standard deviations and p-values of the PC score values from mESCs cultured in control (control mESC) or 2i medium (2i mESC). Two-tailed *t*-test, n=85. (**b**) PC 1 – PC 3 loadings of the control mESCs or 2i mESCs.
